# The Universal Vital Assessment (UVA) score at 6 hours post-resuscitation predicts mortality in hospitalized adults with severe sepsis in Mbarara, Uganda

**DOI:** 10.1371/journal.pgph.0003797

**Published:** 2024-10-22

**Authors:** Megan Null, Mark Conaway, Riley Hazard, Louisa Edwards, Kabanda Taseera, Rose Muhindo, Sam Olum, Amir Abdallah Mbonde, Christopher C. Moore

**Affiliations:** 1 Division of Infectious Diseases and International Health, Department of Medicine, University of Virginia School of Medicine, Charlottesville, Virginia, United States of America; 2 Department of Public Health Sciences, University of Virginia School of Medicine, Charlottesville, Virginia, United States of America; 3 University of Melbourne School of Medicine, Melbourne, Australia; 4 Department of Microbiology, Faculty of Medicine, Mbarara University of Science and Technology, Mbarara, Uganda; 5 Department of Medicine, Faculty of Medicine, Mbarara University of Science and Technology, Mbarara, Uganda; 6 Department of Medicine, Faculty of Medicine, Gulu University, Gulu, Uganda; 7 Department of Neurology, Mayo Clinic Arizona, Scottsdale, Arizona, United States of America; UMass Chan Medical School - Baystate Medical Center, UNITED STATES OF AMERICA

## Abstract

Sepsis is the leading cause of global death with the highest burden found in sub-Saharan Africa (sSA). The Universal Vital Assessment (UVA) score is a validated resource-appropriate clinical tool to identify hospitalized patients in sSA who are at risk of in-hospital mortality. Whether a decrease in the UVA score over 6 hours of resuscitation from sepsis is associated with improved outcomes is unknown. We aimed to determine (1) the association between 6-hour UVA score and in-hospital mortality, and (2) if a decrease in UVA score from admission to 6 hours was associated with improved in-hospital mortality. We analyzed data from participants with severe sepsis aged ≥14 years enrolled at the Mbarara Regional Referral Hospital in Uganda from October 2014 through May 2015. Among 197 participants, the median (interquartile range) age was 34 (27–47) years, 99 (50%) were female and 116 (59%) were living with HIV. At 6 hours, of the 65 participants in the high-risk group, 28 (43%) died compared to 28 (30%) of 94 in the medium-risk group (odds ratio [OR] 0.56, 95% confidence interval [CI] 0.29,1.08, p = 0.086) and 3 (9%) of 33 in the low-risk group (OR 0.13, 95% CI 0.03, 0.42, p = 0.002). In a univariate analysis of the 85 participants who improved their UVA risk group at 6 hours, 20 (23%) died compared to 39 (36%) of 107 participants who did not improve (OR 0.54, 95% CI 0.27–1.06, p = 0.055). In the multivariable analysis, the UVA score at 6 hours (adjusted OR [aOR] 1.26, 95%CI 1.10–1.45, p<0.001) was associated with in-hospital mortality. When adjusted for age and sex, improvement in the UVA risk group over 6 hours was associated with a non-statistically significant 43% decrease in odds of mortality (aOR 0.57, 95%CI 0.29–1.07, p = 0.08). Targeting a decrease in UVA score over 6 hours from admission may be a useful clinical endpoint for sepsis resuscitation in sSA, but this would need to be proven in a clinical trial.

## Introduction

Sepsis is a life-threatening syndrome of organ dysfunction caused by a dysregulated host response to infection. Sepsis is the leading cause of mortality worldwide accounting for 20% of all global deaths, with the highest burden resting on sub-Saharan Africa (sSA) [1]. In 2017, the sepsis incidence in sSA was approximately 1,500 per 100,000 population, which led to an estimated 3.5 million deaths.

Sepsis definitions have changed over time. The currently used Sepsis-3 operational definition was created because the original sepsis definition based on systemic inflammatory response syndrome (SIRS) criteria lacked specificity and sensitivity [2]. Additionally, the previous concept of sepsis as a continuum from sepsis to severe sepsis to shock was flawed. As a result, the new Sepsis-3 definition was derived based on patient data from North America and Europe, which required an increase of ≥2 points in the sequential organ failure assessment (SOFA) score to identify organ dysfunction in the setting of infection. Sepsis-3 also included the derivation of a quick SOFA (qSOFA) score to identify patients at risk for increased mortality from infection in settings where the components of the full SOFA score are not available [2].

Implementing the SOFA score in resource-limited settings is challenging due to often unavailable laboratory testing. The performance of qSOFA in low-income countries (LICs) has varied depending on the population studied [3–5]. Given the differences in critically ill populations, including the high HIV prevalence and constrained resources in LICs compared to those in high-income countries where qSOFA was derived, a Universal Vital Assessment (UVA) score to predict in-hospital mortality in Africa was derived from a large dataset of hospitalized patients from 6 countries in sSA [6]. The UVA score had an overall area under the receiver operating characteristic curve (AUC) of 0.77 for all hospitalized patients and 0.75 for patients with known or suspected infection, both of which outperformed qSOFA. The UVA score has since been validated separately in Gabon, Rwanda, Tanzania, and Uganda, where it also outperformed qSOFA [7–10].

The 2021 Surviving Sepsis Campaign (SSC) International Guidelines for Management of Sepsis and Septic Shock recommend that initial resuscitation should target decreased serum lactate [11]. However, serum lactate testing is not frequently available in hospitals in LICs, and we found there was no association between change in whole blood lactate and mortality from sepsis in Uganda [12]. Given the strong association between UVA scores and mortality, it is possible that targeting a decrease in UVA score as a goal of sepsis resuscitation as an alternative to decreased lactate may be beneficial. Therefore, the objective of this study was to (1) determine the association between 6-hour UVA score and in-hospital mortality, and (2) to determine if a decrease in UVA score from admission to 6 hours was associated with improved in-hospital mortality.

## Materials and methods

We performed a secondary analysis of data collected from participants aged ≥14 years enrolled from the medical emergency ward or adult general medicine ward of Mbarara Regional Referral Hospital (MRRH) in Mbarara, Uganda from October 7, 2014 through May 11, 2015 [12]. We accessed the data for the current analysis from July 15, 2022 through January 1, 2024. MRRH has 600 beds and serves as the main referral center for all the districts in southwestern Uganda as well as neighboring regions of Rwanda and the Democratic Republic of the Congo. It is the teaching hospital for the Mbarara University of Science and Technology Faculty of Medicine.

We determined the sample size for the original study based on the expected mortality difference between participant groups achieving or not achieving a 10% reduction in whole blood lactate concentration. Assuming 25% mortality in the reduction group and 50% in the non-reduction group, with a clinically significant difference of 30%, we calculated a sample size of 199 patients with 90% power and 5% significance level. Allowing for a 10% attrition rate, the final estimated sample size was 218 patients.

Participants were enrolled at admission with severe sepsis defined as: (1) a clinically suspected infection; (2) ≥2 SIRS criteria including an axillary temperature ≥38°C or <36°C, heart rate >90 beats/min, respiratory rate >20 breaths/min, or white blood cell concentration >12,000 cells/μL or <4000 cells/μL; and (3) signs of end-organ dysfunction including a systolic blood pressure of ≤90 mmHg, thrombocytopenia (<100,000 cells/μL), or a Glasgow coma scale (GCS) score <15 [12, 13]. Clinical data including vital signs and point-of-care whole blood lactate were collected at admission and 6 hours later. Recruitment occurred in a consecutive manner throughout the week during the day and evening but not overnight.

Participants missing more than 50% of the clinical variables necessary to compute qSOFA or UVA scores, as well as one participant missing HIV status, were removed from the analysis. We used k-nearest neighbor single imputation to impute remaining missing vital sign values [6, 14]. We imputed only the variables needed for computing scores (i.e., GCS, respiratory rate, systolic blood pressure, temperature, pulse, and oxygen saturation) except HIV status, and calculated qSOFA and UVA scores at admission and 6 hours (S1 Table) [6]. To compute the risk scores at 6 hours, we carried forward both HIV status and the GCS score from admission, as the GCS score was not recalculated at 6 hours. We summarized patient characteristics as frequency with percentage for categorical variables and median with interquartile range (IQR) for continuous variables.

In participants with available mortality outcomes, we calculated in-hospital mortality associated with raw UVA scores. Due to the low frequency of UVA scores ≥10, we combined participants with a UVA score ≥10 into one group. Participants were grouped into predefined low, medium, and high-risk groups based on their UVA score (<2 = low-risk, 2–4 = medium-risk, and >4 = high-risk) and we determined their associated mortality at admission and at 6 hours. We also determined qSOFA-defined sepsis category (qSOFA ≥2 = sepsis) and associated in-hospital mortality for each participant at admission and 6 hours. As we were interested in the change in mortality with improved UVA scores, we computed the odds ratios for mortality for low- and medium-risk groups compared to high-risk referent groups at admission and 6 hours. Improvement was defined as a change in risk group from high to medium or low, or from medium to low. No improvement was defined as no change in risk group over 6 hours. There was one participant with an increase in UVA risk group severity over 6 hours who was removed from this portion of the analysis.

We performed chi-square tests to assess differences in mortality between groups and used logistic regression to assess the ability of UVA and qSOFA scores, as well as change in UVA risk group, to predict mortality. We controlled for age and sex in each logistic regression model. HIV status was not included in our models as it is a component of the UVA score (S1 Table). To determine discriminative performance for in-hospital mortality, we calculated the AUC for each risk score at admission and 6 hours. R Studio version 2023.03.0+386 (Posit Software, PBC) and SPSS software version 28.0.1.1 (IBM SPSS Statistics) were used for analysis.

### Ethics statement

The original study and our secondary analysis were approved by the Faculty Ethics Review Committee and the Institutional Ethical Review Committee at Mbarara University of Science and Technology (#07/09-14) as well as the Institutional Review Board at the University of Virginia. All patients provided formal written informed consent prior to enrollment in the study. If a patient could not provide informed consent, then an accompanying family member or friend provided it for them.

## Results

**Fig 1:**
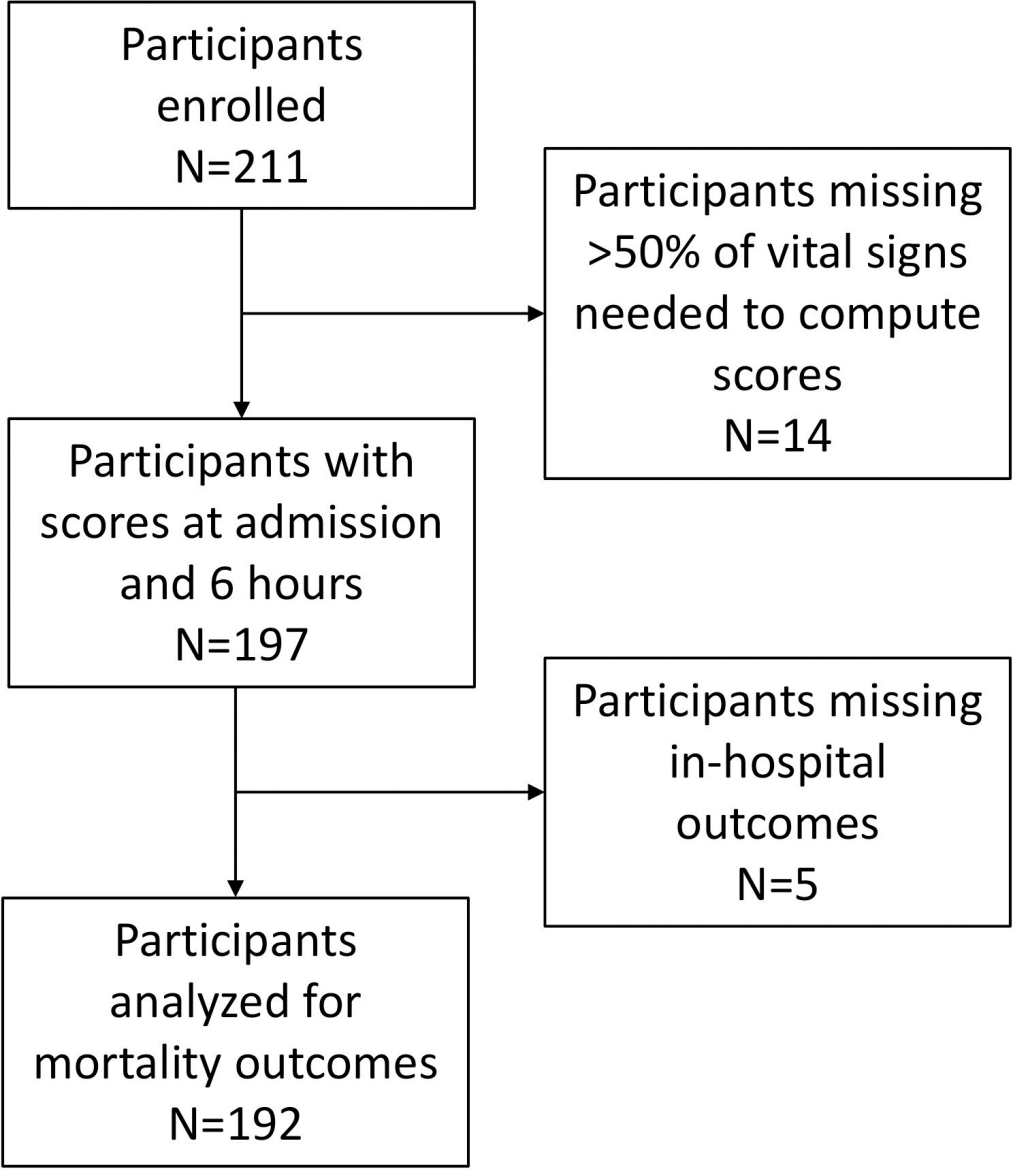
Flow diagram of enrollment and analysis of participants with severe sepsis enrolled at Mbarara Regional Referral Hospital, October 2014 through May 2015.

There were 211 participants enrolled at the time of their admission to hospital. After removing participants with <50% of risk score variables, follow-up data obtained at 6 hours after admission was available from 197 participants (Fig 1). The median (IQR) age was 34 (27–47) years, 99 (50%) were female and 116 (59%) were living with HIV, of whom 70 (60%) were receiving antiretroviral therapy (Table 1). The most common focus of infection was the chest, which was identified in 80 (41%) participants, followed by the gastrointestinal tract, which was found in 54 (27%) participants. Participants spent a median (IQR) of 8 (6–16) hours in the emergency ward. The median length of stay in hospital (IQR) was 6 (3–11) days. The overall in-hospital case fatality rate was 59 (31%) of 192 analyzed participants, and the median (IQR) time to death was 4 (1–8) days.

**Table 1:** Characteristics of participants with severe sepsis enrolled at Mbarara Regional Referral Hospital, October 2014 through May 2015.

Patient characteristics	Missing, n (%)	N = 197*
**Demographics**		
Age (year)	2 (1)	34 (27–47)
Female	1 (<1)	99 (51)
**Clinical parameters**		
Living with HIV	1 (0.3)	116 (59)
CD4+ T-cell concentration (cells/uL)	115 (58)	76 (15–191)
Receiving antiretroviral therapy	0 (0)	70/116 (60)
**Focus of infection**	18 (9)	
Chest		80 (45)
Gastrointestinal		54 (30)
Central nervous system		35 (20)
**Vital signs**		
SBP (mm Hg)	27 (14)	86 (80–94)
DBP (mm Hg)	30 (15)	58 (45.5–60)
MAP (mm Hg)	27 (14)	67 (57–70)
Temperature (°C)	4 (2)	38 (36.5–39)
Heart rate (beats/min)	7 (4)	120 (106–139)
Respiratory rate (breaths/min)	4 (2)	34 (28–44)
Oxygen saturation (%)	8 (4)	93 (88–96)
GCS score	5 (3)	15 (14–15)
MUAC (cm)	3 (2)	22 (19.4–24.7)
**Laboratory values**		
WBC (10^3^ cells/uL)	11 (6)	6.5 (4–12.4)
Hemoglobin (g/dL)	12 (6)	11.1 (7.9–13.1)
Platelets (10^3^ cells/uL)	12 (6)	158 (85–271)
**Resuscitation**		
Received antibiotics	5 (3)	168 (88)
Time to antibiotics (min)	36 (18)	30 (30–80)
Received supplemental oxygen	7 (4)	13 (7)
Total intravenous fluids (L)	0 (0)	1.5 (1.0–2.0)
Time in emergency ward (h)	14 (7)	8 (6–16)
Admission lactate (mmol/L)	0 (0)	3.4 (2.3–5.2)

IQR-interquartile range, CNS-central nervous system, SBP-systolic blood pressure, DBP-diastolic blood pressure, MAP-mean arterial pressure, O_2_-oxygen, GCS-Glasgow coma scale, MUAC-mid-upper arm circumference, WBC-white blood cells

*Values are provided as n (%) or median (interquartile range) from the non-missing values for each variable.

**Fig 2:**
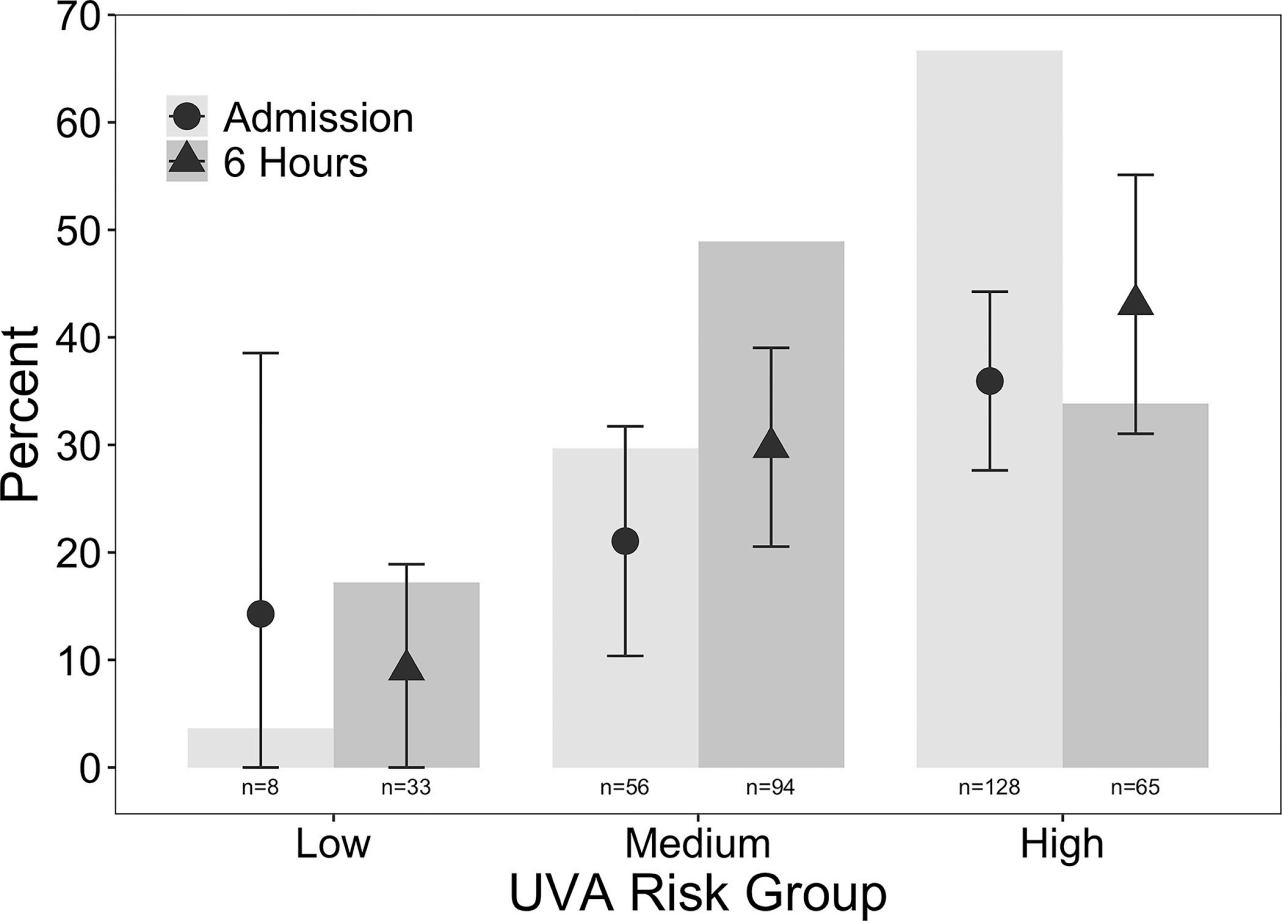
The frequency (%) of participants in each UVA score risk group within the total study population displayed in bars, and the associated in-hospital mortality rates, with 95% confidence intervals, depicted at admission (circles) and at 6 hours post-admission (triangles) among participants admitted with severe sepsis to Mbarara Regional Referral Hospital from October 2014 to May 2015.

**Fig 3:**
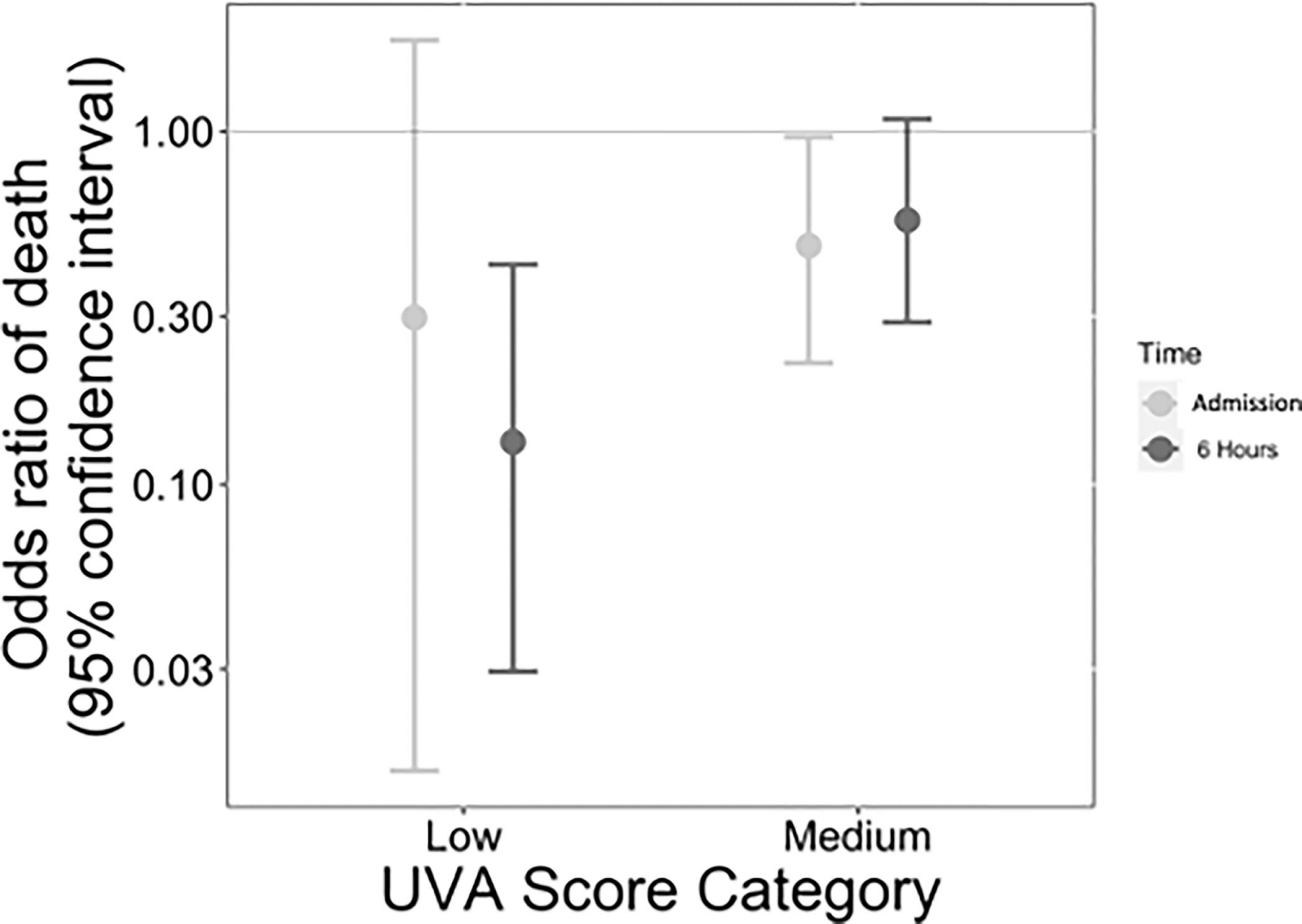
Adjusted odds ratios with 95% confidence intervals for in-hospital mortality associated with UVA score low- and medium-risk groups at admission and 6 hours post-admission, compared to the high-risk group among participants admitted with severe sepsis to Mbarara Regional Referral Hospital between October 2014 and May 2015.

Of the 192 participants with known mortality outcomes, higher UVA raw scores and risk group severity were associated with increased mortality (Fig 2, S1 Fig). At admission, of the 128 participants in the high-risk group, 46 (36%) died compared to 12 (21%) of 57 in the medium-risk group (odds ratio [OR] 0.48, 95% confidence interval [CI] 0.22–0.97, p = 0.047) and 1 (14%) of 7 in the low-risk group (OR 0.30, 95% CI 0.02–1.81, p = 0.27). At 6 hours, of the 65 participants in the high-risk group, 28 (43%) died compared to 28 (30%) of 94 in the medium-risk group (OR 0.56, 95% CI 0.29–1.08, p = 0.086) and 3 (9%) of 33 in the low-risk group (OR 0.13, 95% CI 0.03–0.42, p = 0.002) (Table 2 and Fig 3).

**Table 2:** Mortality according to UVA risk groups at admission and 6 hours among participants admitted with sepsis to Mbarara Regional Referral Hospital, October 2014 through May 2015.

**In-hospital mortality by admission UVA risk group**				
	Low N = 7	Medium N = 57	High N = 128	Total N = 192
Survived	6 (86%)	45 (79%)	82 (64%)	133 (69%)
Died	1 (14%)	12 (21%)	46 (36%)	59 (31%)
Chi-square 5.03, p = 0.081				
**In-hospital mortality by 6-hour UVA risk group**				
	Low N = 33	Medium N = 94	High N = 65	Total N = 192
Survived	30 (91%)	66 (70%)	37 (57%)	133 (69%)
Died	3 (9%)	28 (30%)	28 (43%)	59 (31%)
Chi-square 11.95, p = 0.003				

**Fig 4:**
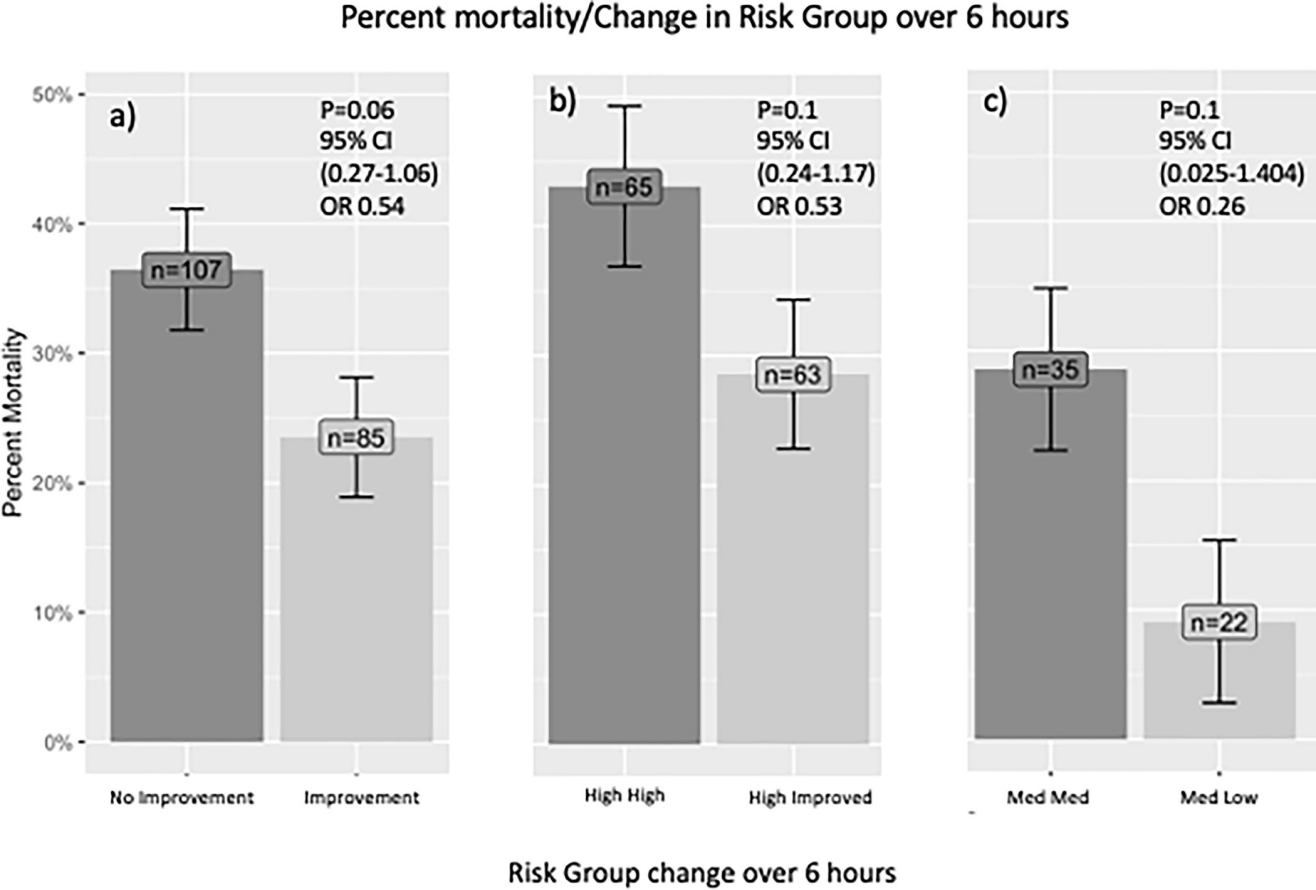
In-hospital mortality associated with improvement status in UVA score risk groups over 6 hours from admission among participants admitted with severe sepsis to Mbarara Regional Referral Hospital, October 2014 through May 2015; A) in-hospital mortality according to improvement status for all participants; B) in-hospital mortality for participants in the UVA high-risk group at admission who remained high-risk (High High) and for those who improved (High Improved) at 6 hours; and C) in-hospital mortality for participants in the UVA medium-risk group at admission who remained medium-risk (Med Med) and for those who improved (Med Low) at 6 hours.

**Fig 5:**
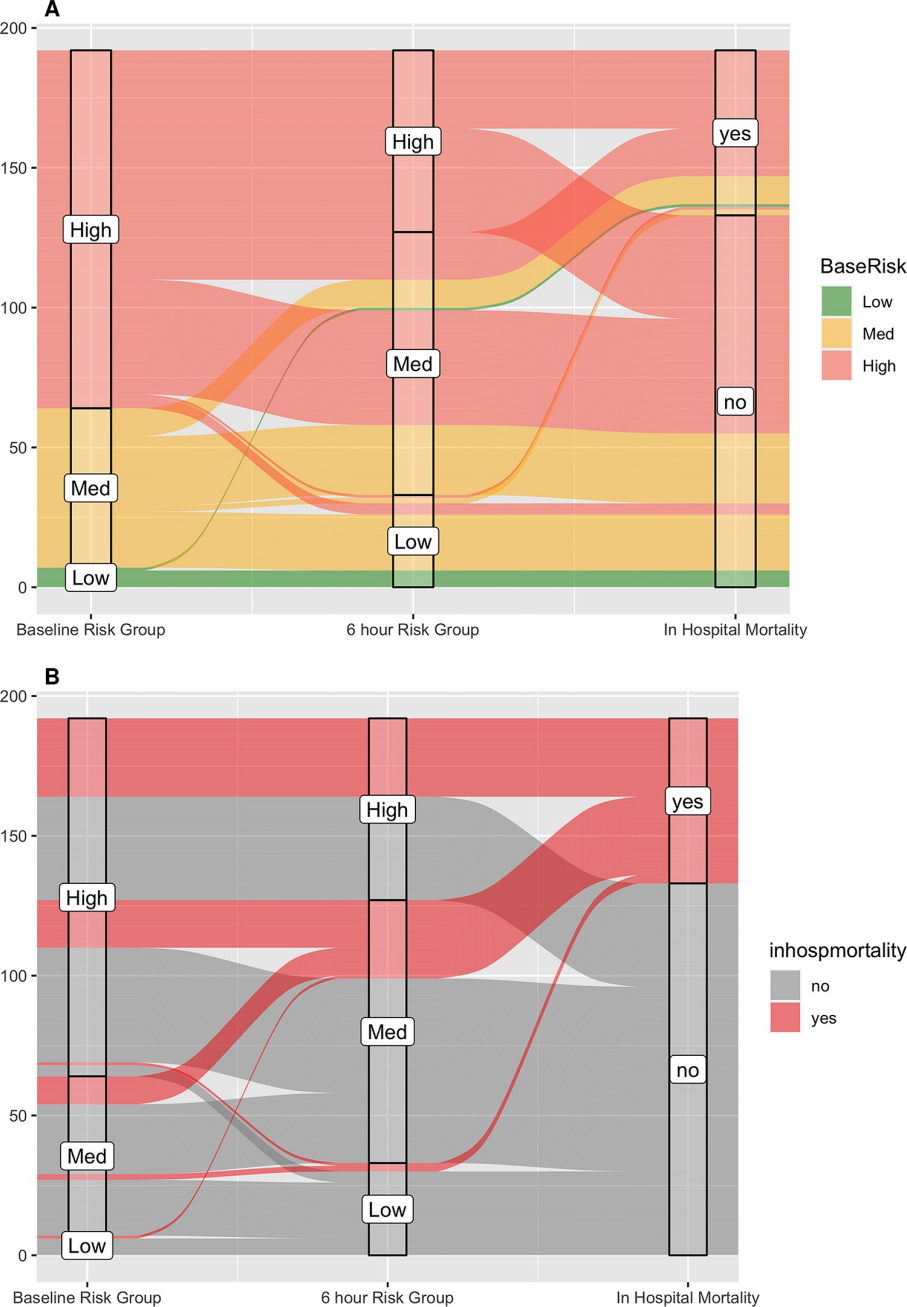
Alluvial plots of the trajectory of participants within each UVA score risk group as they progressed from admission through 6 hours to in-hospital mortality outcomes color-coded by A) admission UVA risk group, and B) mortality outcome.

Of the 85 participants with improvement in their UVA risk group at 6 hours, 20 (23%) died compared to 39 (36%) of 107 participants who did not improve (OR 0.54, 95% CI 0.27–1.06, p = 0.055) (Fig 4A). Of the 63 participants in the high-risk group at admission who were improved at 6 hours, 18 (29%) died compared to 28 (43%) of 65 participants who did not improve (OR 0.53, 95%CI 0.24–1.17, p = 0.089) (Table 3 and Fig 4B). Of the 22 participants in the medium-risk group at admission who were improved at 6 hours, 2 (9%) died compared to 10 (29%) of 35 who did not improve (OR 0.26, 95%CI 0.025–1.40, p = 0.095) (Fig 4C). Fig 5 displays the trajectory of participants within each UVA risk group as they progressed from admission through 6 hours to in-hospital mortality outcomes.

**Table 3:** Mortality according to change in UVA risk group and qSOFA sepsis category from admission to 6-hours among participants admitted with sepsis to Mbarara Regional Referral Hospital, October 2014 through May 2015.

Mortality according to admission and 6-hour UVA risk group and qSOFA sepsis category				
		UVA risk group at 6 hours		
		Low	Medium	High
Admission UVA risk group	Low	0/6 (0%)	1/1 (100%)	0/0 (0%)
	Medium	2/22 (9%)	10/35 (29%)	0/0 (0%)
	High	1/5 (20%)	17/58 (29%)	28/65 (43%)
		qSOFA sepsis category at 6 hours		
		No Sepsis	Sepsis	
Admission qSOFA sepsis category	No Sepsis	7/19 (37%)	1/6 (17%)	
	Sepsis	7/28 (25%)	44/139 (32%)	

Of the 139 participants with qSOFA-defined sepsis at admission and 6 hours, 44 (32%) died compared to 7 (25%) of 28 with sepsis at admission but not at 6 hours (OR 0.72, 95%CI 0.24–1.93, p = 0.49). Of the 19 participants who did not have sepsis at admission or at 6 hours, 7 (37%) died compared to 1 (17%) of 6 participants with sepsis at 6 hours who did not have sepsis at admission (OR 0.36, 95%CI 0.01–4.24, p = 0.36) (Table 3).

At admission and 6 hours, the AUC was 0.63 and 0.65 for the UVA score, and 0.52 and 0.51 for qSOFA (S2 Fig). In the multivariable analysis, the UVA score at admission (adjusted OR [aOR] 1.19, 95%CI 1.05–1.36, p = 0.007) and at 6 hours (aOR 1.26, 95%CI 1.10–1.45, p<0.001) was associated with in-hospital mortality. When adjusted for age and sex, improvement in the UVA risk group over 6 hours was associated with a non-statistically significant 43% decrease in odds of mortality when compared to those who did not improve (aOR 0.57, 95%CI 0.29–1.07, p = 0.08) (Table 4). In a separate multivariable model, there was no independent association between improvement in UVA score and clinical interventions including administration of intravenous fluids, antibiotics, or supplemental oxygen; or clinical variables including CD4+ T cell concentration or lactate concentration.

**Table 4:** Baseline logistic regression models of mortality with and without the inclusion of admission and 6 hour UVA and qSOFA scores, and UVA score improvement from participants admitted with sepsis to Mbarara Regional Referral Hospital, October 2014 through May 2015.

Variable	Base model		Base + admission UVA		Base + 6-hour UVA		Base + admission qSOFA		Base + 6-hour qSOFA		Base + UVA score improvement	
	OR (95%CI)	p	OR (95% CI)	p	OR (95% CI)	p	OR (95%CI)	p	OR (95%CI)	p	OR (95% CI)	p
Age	1.00 (0.99–1.02)	0.71	1.00 (0.99–1.02)	0.66	1.01 (0.99–1.03)	0.45	1.00 (0.98–1.02)	0.77	1.00 (0.99–1.02)	0.70	1.00 (0.99–1.02)	0.65
Male	0.96 (0.52–1.79)	0.9	0.79 (0.41–1.51)	0.48	0.79 (0.41–1.52)	0.48	0.94 (0.50–1.76)	0.84	0.94 (0.50–1.76)	0.85	0.91 (0.48–1.70)	0.76
UVA	---	---	1.19 (1.05–1.36)	0.007	1.26 (1.10–1.45)	<0.001	---	---	---	---	0.57 (0.29–1.07)	0.08
qSOFA	---	---	---	---	---	---	1.18 (0.70–2.00)	0.53	1.21 (0.77–1.91)	0.41	---	---
AUC	0.48		0.64		0.66		0.54		0.54		0.56	

AUC, area under the receiver operating characteristic curve; OR, odds ratio; CI, confidence interval

## Discussion

In this study, we investigated the association of clinical risk scores with in-hospital mortality after the first 6 hours of resuscitation in adults with severe sepsis in Uganda. We found that a patient’s UVA score at both admission and 6 hours was a strong predictor of mortality and outperformed the qSOFA score. Sepsis remains a significant burden in LICs where limited healthcare resources and infrastructure present unique challenges in the timely recognition and management of sepsis. Our data suggest that UVA scores can aid in the early determination of the risk of sepsis-related mortality and an improvement in the UVA score over 6 hours could provide a useful endpoint for sepsis resuscitation.

Available data on sepsis management of patients across the age spectrum in LICs suggest that the reasons for high associated case fatality rates are complex and due in part to human and material resource limitations, antimicrobial resistance, and a lack of available critical care facilities [15–17]. Clearly, improved sepsis management in these settings is urgently needed to reduce mortality [18]. Although clinicians in LICs may have limited access to technological infrastructure and clinical laboratories, the UVA score can be calculated using readily available clinical parameters and bedside observations. Therefore, the implementation of UVA score monitoring could be accomplished in any clinical setting regardless of available resources.

The optimal clinical management of sepsis in sSA has not been determined. However, UVA scores and clinical outcomes could potentially be improved by timely administration of appropriate antimicrobial therapy [19]. The high prevalence of *Mycobacterium tuberculosis* (*Mtb*) bloodstream infection as a cause of sepsis has significant implications for appropriate antimicrobial therapy in sSA [20]. Despite the high prevalence of *Mtb* bloodstream infection, current WHO guidelines for the management of acutely ill patients in LICs recommend rapid administration of broad-spectrum antimicrobials to treat common Gram positive and Gram negative bacteria, but not *Mtb* [21–23]. Higher UVA scores are associated with the clinical and immune phenotypes of *Mtb* bloodstream infection and could aid decisions on empiric anti-*Mtb* therapy [24]. Fluid administration could potentially improve UVA scores and sepsis outcomes [11, 19]. However, without appropriate monitoring equipment, both restrictive and liberal fluid resuscitation could worsen outcomes for sepsis patients, despite a decrease in UVA risk score [15, 25, 26]. Our study did not find any clear associations between these variables and improvement in UVA risk group; however, patients who are perceived to be the most severely ill may receive more interventions, which are often futile [27].

The SSC guidelines recommend immediate resuscitation and administration of empirical antimicrobial therapy within one hour of recognition of sepsis or septic shock [11]. Implementation of these guidelines in LICs is a significant challenge. A survey of 185 African Hospitals conducted in 2009 found that less than 1.5% of the hospitals surveyed had the resources available to consistently implement the SSC guidelines or any of their sepsis resuscitation and management bundles [28]. Among the most notable deficits was the inconsistent supply or lack of oxygen, fluids, broad-spectrum antibiotics, essential disposables, and monitoring equipment [22]. In our study, due to resource constraints, only 7% of participants received supplemental oxygen. Modification of existing guidelines could ensure that available resources are deployed appropriately according to the latest clinical evidence. The inclusion of the UVA score to evaluate the severity and trajectory of sepsis could expedite care and guide allocation of limited available resources to those patients who would benefit the most.

This study had limitations. Our analyses were based on a relatively small sample size from a single institution. Additionally, our participants were enrolled based on Sepsis-2 criteria (SIRS), and our analysis subjected them to a second categorization for the same syndrome (UVA and qSOFA). There was no follow-up GCS score evaluated at 6 hours, and while it is not expected that cognition would change dramatically over 6 hours, the UVA scores would have been more accurate if GCS score had been reassessed. For example, assuming that participants whose GCS score improved over 6 hours were more likely to survive, it is possible that we have underestimated the association between UVA score and mortality at 6 hours. We also had limited knowledge of the interventions administered during the 6-hours from admission, thus we could not provide a complete assessment of factors possibly associated with a decrease in UVA score. Finally, we could only calculate risk scores at admission and 6 hours later. We have previously shown that UVA score calculation at 72 hours after admission was not a useful measure of future in-hospital mortality in Rwanda [10]. However, it is possible that calculating the UVA score before 6 hours after admission, or after 6 hours but before 72 hours could be clinically useful. It is also possible that more frequent serial monitoring of the UVA score could assist with patient management.

## Conclusions

Among hospitalized patients aged ≥14 years with severe sepsis at a regional referral hospital in Uganda, the UVA score at admission and 6 hours provided enhanced in-hospital mortality prediction over a single UVA assessment at admission and qSOFA sepsis determination. An improvement in UVA risk group over 6 hours was associated with a decrease in in-hospital mortality. Further prospective studies are needed to understand the clinical variables and interventions associated with a decrease in UVA risk scores and whether it is beneficial to reduce the UVA score for improved patient outcomes. Follow-up studies of longitudinal UVA score monitoring could include a cluster randomized trial. The UVA score could also be assessed to guide the administration of adjunctive therapies including intravenous fluids and corticosteroids.

## Supporting information

S1 Fig The frequency (%) of participants at each UVA risk score within the total study population displayed in bars, and the associated in-hospital mortality rates, with 95% confidence intervals, depicted at admission (circles) and at 6 hours post-admission (triangles) among participants admitted with severe sepsis to Mbarara Regional Referral Hospital from October 2014 to May 2015. (TIFF)

S2 Fig Receiver operating characteristic curves for the discriminative performance of UVA and qSOFA scores at admission and 6 hours for in-hospital mortality among participants admitted with severe sepsis to Mbarara Regional Referral Hospital, October 2014 through May 2015. (TIFF)

S1 Table Universal Vital Assessment (UVA) and quick sequential organ failure assessment. (DOCX)

S1 Data Dataset. (XLSX)
