# Supplementary figures and images for: The Universal Vital Assessment (UVA) score at 6 hours post-resuscitation predicts mortality in hospitalized adults with severe sepsis in Mbarara, Uganda
Source: PLOS Glob Public Health. 2024 Oct 22;4(10):e0003797. doi: 10.1371/journal.pgph.0003797 (PMC11495629; doi:10.1371/journal.pgph.0003797)

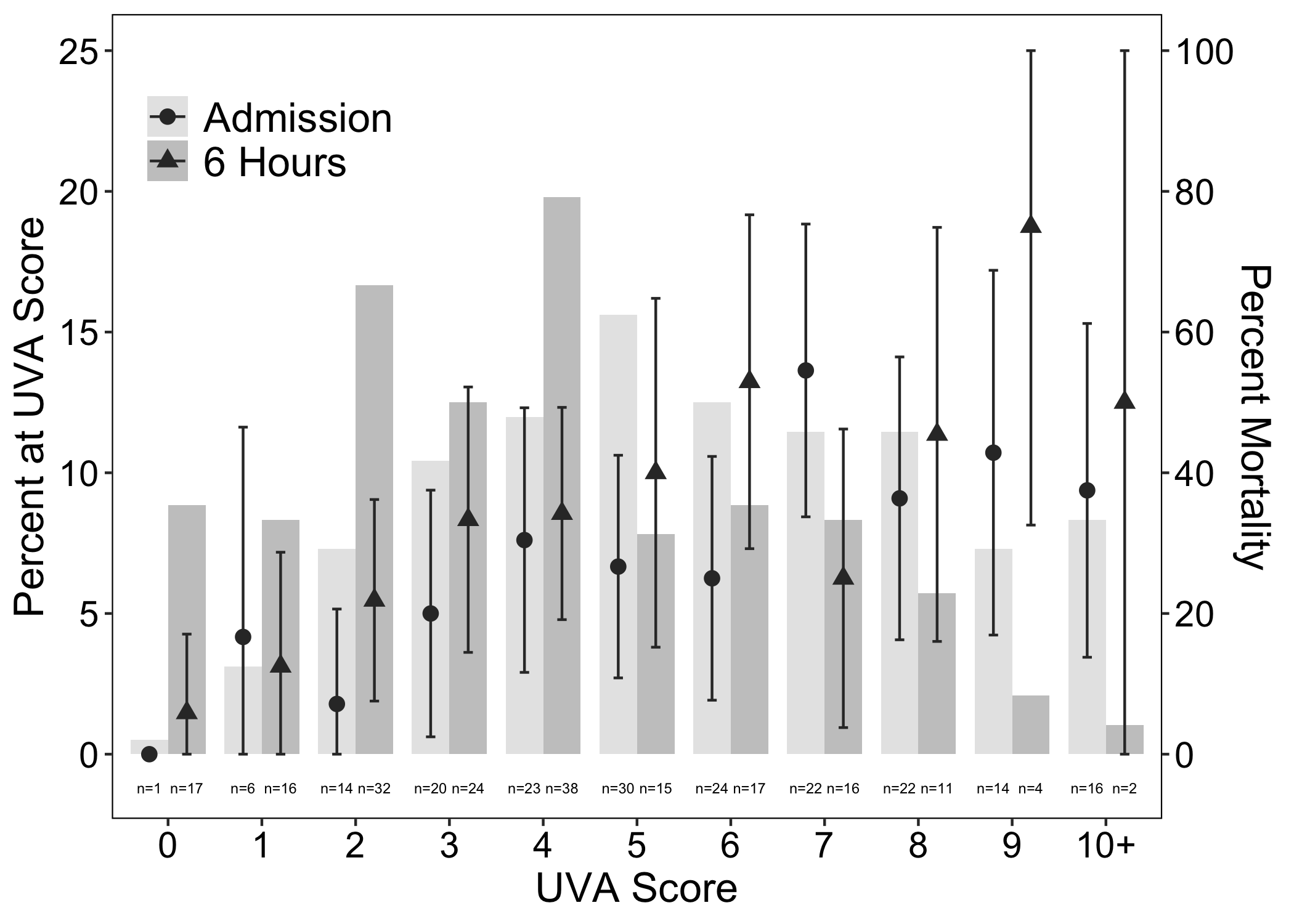

Supplement: S1 Fig — (TIFF) [file pgph.0003797.s001.tiff]

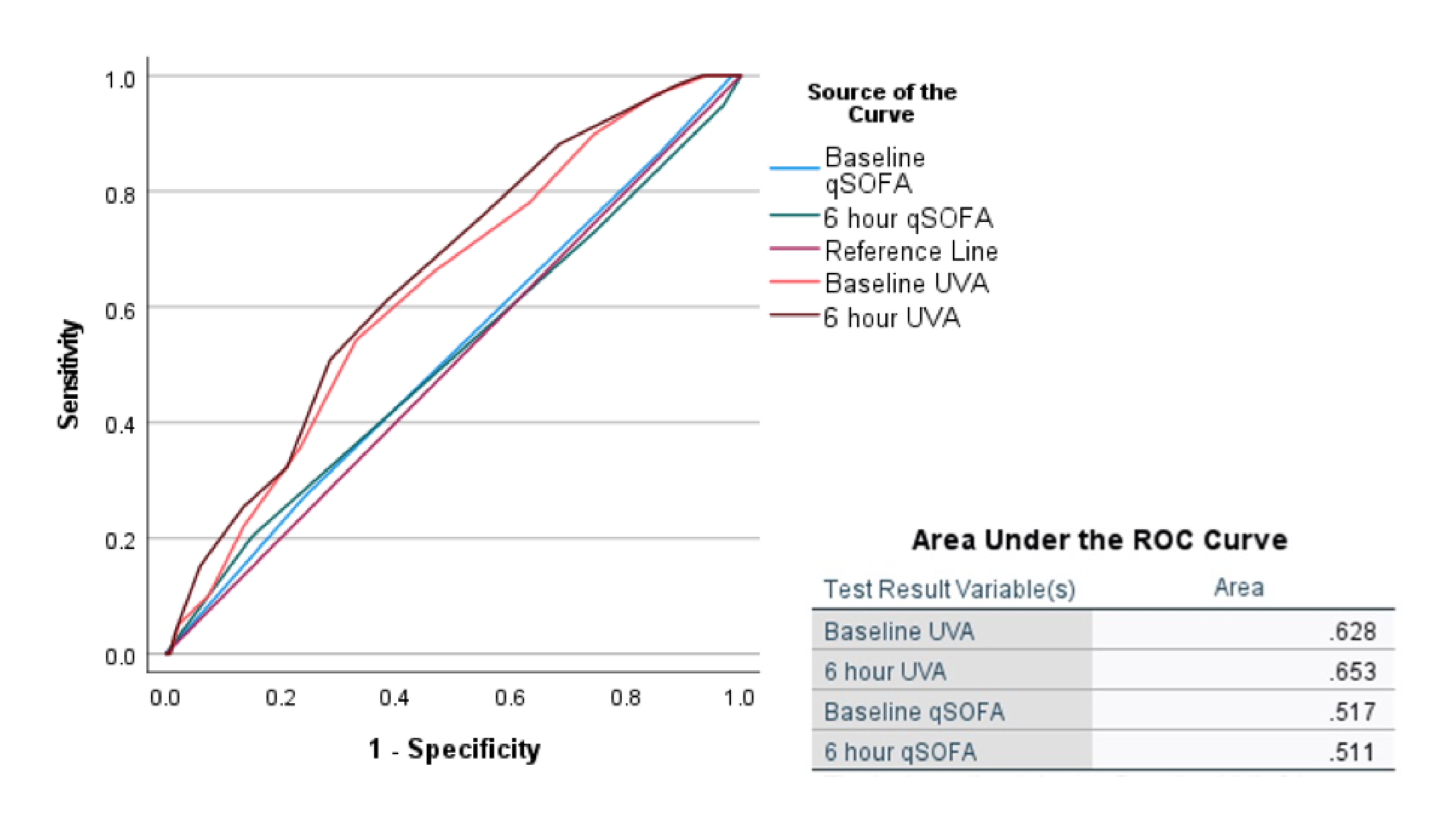

Supplement: S2 Fig — (TIFF) [file pgph.0003797.s002.tiff]
